# Mode of renal replacement therapy determines endotoxemia and neutrophil dysfunction in chronic kidney disease

**DOI:** 10.1038/srep34534

**Published:** 2016-10-04

**Authors:** Sandra Lemesch, Werner Ribitsch, Gernot Schilcher, Walter Spindelböck, Hildegard Hafner-Gießauf, Gunther Marsche, Lisa Pasterk, Doris Payerl, Bianca Schmerböck, Monika Tawdrous, Alexander R. Rosenkranz, Philipp Stiegler, Gerd Kager, Seth Hallström, Karl Oettl, Katharina Eberhard, Angela Horvath, Bettina Leber, Vanessa Stadlbauer

**Affiliations:** 1 Department of Internal Medicine, Division of Gastroenterology and Hepatology, Medical University of Graz, Graz, Austria; 2 Department of Internal Medicine, Clinical Division of Nephrology, Medical University of Graz, Graz, Austria; 3 Intensive Care Unit, Department of Internal Medicine, Medical University of Graz, Graz, Austria; 4 Institute of Experimental and Clinical Pharmacology, Medical University of Graz, Graz, Austria; 5 Institute of Physiological Chemistry, Center of Physiological Medicine, Medical University of Graz, Graz, Austria; 6 Department of Surgery, Division of Transplantation Surgery, Medical University of Graz, Graz, Austria; 7 Core Facility Computational Bioanalytics, Center for Medical Research, Medical University of Graz, Graz, Austria

## Abstract

Bacterial infection and sepsis are common complications of chronic kidney disease (CKD). A vicious cycle of increased gut permeability, endotoxemia, inadequate activation of the innate immune system and resulting innate immune dysfunction is hypothesized. We assessed endotoxemia, neutrophil function and its relation to oxidative stress, inflammation and gut permeability in patients with CKD grade 3–5 without renal replacement therapy (CKD group, n = 57), patients with CKD stage 5 undergoing haemodialysis (HD, n = 32) or peritoneal dialysis (PD, n = 28) and patients after kidney transplantation (KT, n = 67) in a cross-sectional observational study. In HD patients, endotoxin serum levels were elevated and neutrophil phagocytic capacity was decreased compared to all other groups. Patients on HD had a significantly higher mortality, due to infections during follow up, compared to PD (p = 0.022). Oxidative stress, neutrophil energy charge, systemic inflammation and gut permeability could not completely explain these differences. Our findings suggest that dialysis modality and not renal function per se determine the development of neutrophil dysfunction and endotoxemia in CKD-patients. HD patients are particularly prone to neutrophil dysfunction and endotoxemia whereas neutrophil function seems to improve after KT. Multi-target approaches are therefore warranted to improve neutrophil function and potentially reduce the rate of infections with patients undergoing haemodialysis.

Chronic kidney disease (CKD) has a prevalence of 10% in the general population and up to 20% in high risk groups, such as patients with diabetes^1^. Despite the widespread availability of renal replacement therapy in the western world, mortality of patients with end-stage renal disease (ESRD) remains high. Bacterial infection and sepsis account for up to 20% of deaths in ESRD patients and are a major cause of morbidity and hospitalization^2^,^3^,^4^,^5^,^6^,^7^. A defect of the immune response is thought to partly account for this observation. In ESRD the ability of neutrophils to phagocytize and kill bacteria is impaired. However, the reasons for the observed dysfunction are still not well understood. Iron overload, zinc deficiency, increased intracellular calcium, anaemia, malnutrition, time on dialysis, uremia and adverse effects of the dialysis treatment itself have been linked to neutrophil dysfunction^8^. On the other hand the immune system in patients with ESRD seems to be chronically activated, leading to increased neutrophil oxidative burst and increased levels of inflammatory markers (cytokines, acute-phase-proteins) which are strong predictors of mortality^9^,^10^,^11^,^12^,^13^. Neutrophil activation and dysfunction are probably caused by endotoxemia^14^, a well-recognized phenomenon in ESRD. Bacterial translocation across an impaired gastrointestinal barrier might be a source for endotoxin^15^,^16^,^17^,^18^,^19^,^20^. Gut stunning may explain such a disruption of the gut-mucosal barrier^21^. In addition, if present in the blood circulation, endotoxin (as measured by surrogates such as soluble cluster of differentiation 14 (sCD14)) is also an independent predictor for mortality in ESRD^22^,^23^.

We therefore aimed to assess the relationship between endotoxemia, neutrophil function, oxidative stress, inflammation and gut permeability in CKD patients including CKD stages 3–5, in patients with ESRD undergoing haemodialysis (HD) or peritoneal dialysis (PD) and after kidney transplantation (KT).

## Results

### Patient characteristics

**Figure 1:**
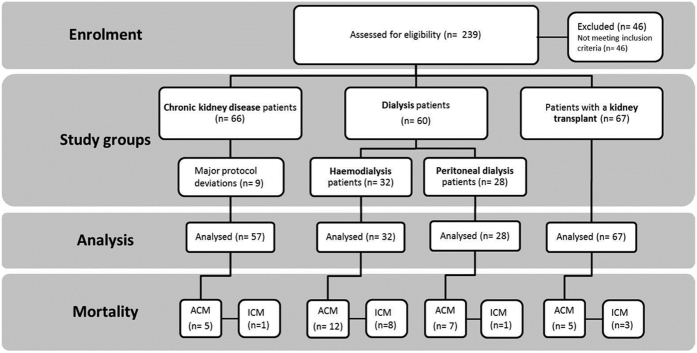
Study evaluation according to TREND. ACM all-cause mortality, ICM infectious-cause mortality.

We enrolled 184 CKD patients (57 patients comprising CKD stages 3–5, 60 ESRD patients undergoing dialysis treatment and 67 patients after KT) and 25 healthy controls (Fig. 1). Of the 57 CKD patients stage 3–5, 17 patients had an estimated glomerular filtration rate (eGFR) between 30–59 ml/min/1.73 m^2^, 29 had an eGFR between 15–29 ml/min/1.73 m^2^ and 11 had an eGFR <15 ml/min/1.73 m^2^. Of 67 patients after KT 53 patients had an eGFR between 30–59 ml/min/1.73 m^2^ and 14 had an eGFR between 15–29 ml/min/1.73 m^2^. Of the 60 dialysis patients 32 were on HD and 28 on PD. All HD patients stayed haemodynamically stable throughout their treatment sessions. Patient groups and healthy controls were comparable in age, body mass index (BMI) and gender distribution. Patient characteristics are presented in Table 1.

### Endotoxemia and neutrophil dysfunction in dialysis patients

In the dialysis group, endotoxin was significantly increased compared to patients with CKD stage G 3–5, patients after KT and controls (p = 0.015). The levels of lipopolysaccharide binding protein (LBP) and sCD14 were increased in all patient groups compared to controls (Table 2). In dialysis patients, neutrophil phagocytosis was significantly decreased to 76% of normal levels. Only a small percentage of neutrophils did not show any phagocytic activity at all. The percentage of inactive neutrophils was around 1% in healthy controls and significantly increased to 2.6% and 2.2% in dialysis patients and patients after KT (p = 0.001 and p = 0.002 respectively), whereas there was no difference compared to controls for CKD stages G3–5 patients. Age and comorbidities did not influence neutrophil phagocytosis. No relevant differences in resting burst, priming or *E. coli* stimulated burst of neutrophils were observed between CKD stages G3–5, dialysis and KT. The absolute neutrophil count was similar in patients and healthy controls. However, the neutrophil/lymphocyte ratio was significantly elevated in all patient groups compared to controls. Gut permeability as measured by diamine-oxidase (DAO) was elevated in CKD stage G3–5, dialysis patients and after KT. Neutrophil energy charge was unaltered in all groups (Table 2). Oxidative stress as measured by advanced oxidation protein products (AOPPs) and carbonylated proteins (CP) was significantly increased in all patient groups. Also, albumin oxidation was significantly altered as shown by a decrease in the reduced albumin fraction (human mercaptalbumin, HMA) and an increase in the oxidized fractions (human non-mercaptalbumin 1 and 2, HNA1 and HNA2; Table 2). The cytokine and cytokine receptor panel (Interleukin (IL)6, sIL6-receptor (sIL6 R), IL8, IL10, tumor necrosis factor (TNF)α, TNFR1, TNFR2) showed large variations but were also elevated in all patient groups compared to healthy controls (Table 2). C reactive protein (CRP) was not elevated in any patient group compared to controls (Table 1).

### Differences in endotoxemia and neutrophil function with regard to dialysis modality

**Figure 2:**
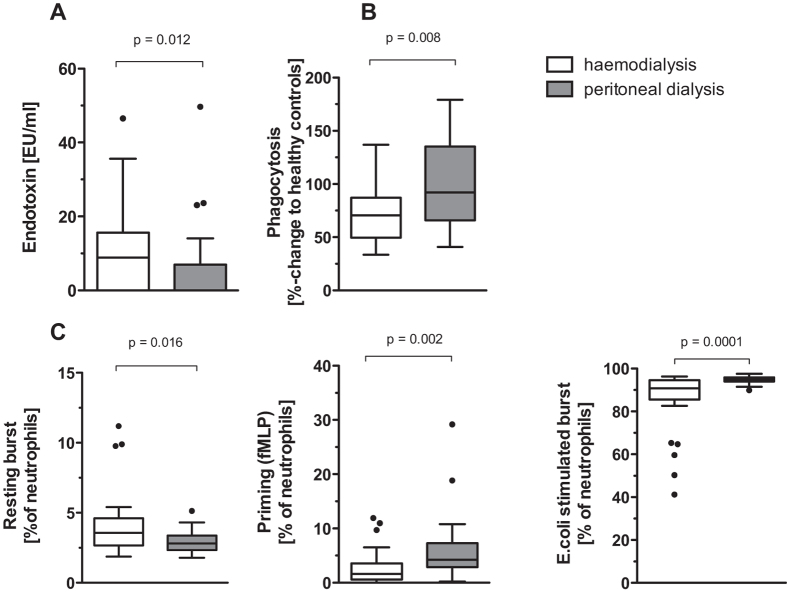
Comparison between haemodialyis and peritoneal dialysis patients. (**A**) endotoxin levels, (**B**) neutrophil phagocytosis (**C**) neutrophil oxidative burst.

HD and PD patients did not differ in age, BMI, renal function or serum albumin, but HD patients had a longer dialysis vintage and a lower residual diuresis compared to PD patients (Table 1). Endotoxin was significantly higher and neutrophil phagocytosis was significantly lower in patients undergoing HD compared to PD (Fig. 2A,B). Age and comorbidities did not influence neutrophil phagocytosis. The absolute neutrophil count, the neutrophil/lymphocyte ratio and the percentage of inactive neutrophils was similar within HD and PD. LBP, sCD14, DAO and energy charge were comparable between HD and PD patients (Table 2). Neutrophil resting burst was significantly higher in HD patients, whereas burst after a mild (fMLP priming) or high (*E. coli*) stimulus was significantly reduced (Fig. 2C). AOPPs and CP were higher in HD than in PD patients, whereas albumin oxidation showed only a trend towards lower levels of HMA and higher levels of HNA 1 and HNA 2. Cytokine profiles showed lower IL10 but higher TNFR1 and TNFR2 levels in HD patients. CRP was elevated in HD patients compared to PD patients (Table 2). The type of vascular access in HD patients had no influence on endotoxemia, neutrophil function, or oxidative stress, but patients with a central venous catheter had higher CRP, sIL6R and TNFR1 levels (Table 3).

### Influence of kidney function on endotoxemia and neutrophil function

Within the group of CKD patients, no difference in BMI, gender or serum albumin levels was found between different CKD stages; however, CKD patients with an eGFR >30 ml/min/1.73 m^2^ were younger than patients with an eGFR <29 ml/min/1.73 m^2^ and <15 ml/min/1.73 m^2^ (Table 4). The stage of CKD had no influence on endotoxin levels, LBP, sCD14, DAO, neutrophil phagocytosis, absolute neutrophil count, neutrophil/lymphocyte ratio, percentage of inactive neutrophils, energy charge, AOPPs and CP (Table 4). Neutrophil resting burst significantly increased with decreasing eGFR. In addition, HMA decreased and HNA1 and HNA2 increased significantly with the decreasing eGFR. In the cytokine panel, IL8 and TNFR1 were significantly higher in patients with an eGFR <15 ml/min/1.73 m^2^ (Table 4).

The same pattern was seen in patients after KT: eGFR was not associated with changes in endotoxin, LBP, sCD14, DAO, neutrophil oxidative burst, absolute neutrophil count, neutrophil/lymphocyte ratio, percentage of inactive neutrophils, energy charge, AOPPs or CP. However, in patients after KT with an eGFR <30 ml/min/1.73 m^2^, neutrophil phagocytosis was significantly impaired, whereas in KT patients with an eGFR >30 ml/min/1.73 m^2^ neutrophil phagocytosis remained unaffected. Immunosuppressive therapy was not different between these groups (data not shown). Furthermore, KT patients with an eGFR <30 ml/min/1.73 m^2^ had lower HMA and higher HNA1 and HNA2 levels as well as higher IL6, IL8, TNFR1 and TNFR2 levels.

### Haemodialysis was associated with higher mortality due to infections

In total 29 out of 184 patients died during the follow up period (median 39 months, range 30–44). All-cause mortality was lower in CKD and KT patients ((5/57 patients) and (5/67 patients)) compared to dialysis patients ((19/60 patients), p = 0.012). There was no difference in all-cause mortality between HD and PD patients (Table 1).

**Figure 3:**
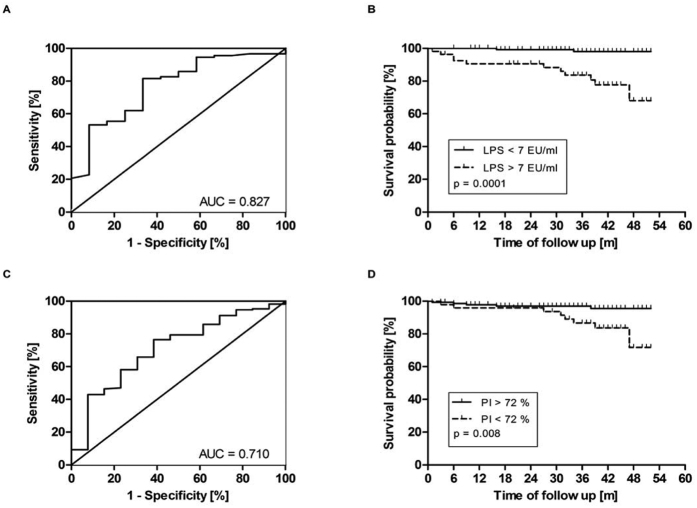
Survival analyses of CKD and ESRD patients. ROC curves (left) were used to determine the cut-off level of endotoxin (**A**) and phagocytosis (PI) (**C**). Both Kaplan Maier curves (right) show that patients with increased endotoxin levels (LPS) (**B**) or reduced phagocytosis (**D**) die more often from infection.

Mortality due to infections was highest in HD patients (8/12 deaths; 67%) and significantly lower in PD (1/5 deaths; 20%) patients (p = 0.022). Regarding the impact of vascular access on mortality and infections, the absolute numbers were too low to draw any meaningful statistical conclusion (Table 3). Endotoxin and neutrophil phagocytosis were predictive for mortality due to infection. Endotoxin levels were considerably higher in patients who later developed fatal infection compared to patients who died from other causes (15.3 (6.5, 29.3) versus 0.0 (0.0, 10.8), p = 0.025, [EU/ml]) and were able to predict mortality from infections (Fig. 3; ROC curve: AUC = 0.827, sensitivity = 0.85, specificity = 0.75, LPS cut-off = 7.1 [EU/ml]). Baseline neutrophil phagocytic capacity was significantly reduced in patients who died from infections as compared to those who died from other causes (70.1 (53.9, 89.9) versus 94.5 (73.3, 145.5), (p = 0.033), [% change of healthy controls]).

All other studied markers did not differ between patients who died from infections as compared to patients who died from other causes.

## Discussion

In this cross-sectional study we demonstrate that patients on haemodialysis exhibit higher levels of endotoxemia and pronounced neutrophil dysfunction compared to PD patients, CKD stage G3–5 patients as well as patients after KT. Differences in oxidative stress, systemic inflammation, neutrophil energy status or gut permeability did not explain these findings.

In ESRD undergoing renal replacement therapy the occurrence of systemic endotoxemia is a well-recognized phenomenon. Potential sources of endotoxin in patients with ESRD are the use of non-ultrapure (contaminated) water as dialysate or bacterial translocation across an impaired mucosal gut barrier. Nowadays, due to strict regulations of dialysate composition, the risk of endotoxemia due to contaminated dialysate has been minimized^24^. In healthy subjects, small amounts of endotoxin regularly pass the mucosal barrier and enter the portal circulation^25^. The liver normally protects the systemic circulation from spill-over of bacteria and their products, mostly originating from the gut^26^. The liver is therefore the major site for endotoxin clearance in the body^25^. The reasons for the presence of high systemic levels of endotoxin in ESRD, where the liver should have a normal function, remain unclear. Possible explanations are that the degree of translocation exceeds the scavenging capacity of the liver or that the renal insufficiency attenuates the endotoxin clearance of the liver.

Systemic endotoxemia is associated with adverse outcomes in several diseases^27^. In ESRD the presence of sCD14 as a surrogate parameter of endotoxin load has been described as an independent predictor for mortality^22^,^23^. We found that increased endotoxin levels at baseline were predictive for death due to infections during follow up. Infection related mortality was lower in PD patients who share the same degree of renal impairment. Other studies have shown a higher risk for infections in PD patients^28^ or similar overall infection rates between these two dialysis modalities, but mortality was not reported^29^. Since the fatality of an infection is not only influenced by the host or the dialysis modality but also by several other factors, such as access to medical care and standards in patient care, further larger studies are needed to clarify these diverging observations.

In our study neutrophil phagocytic capacity was only impaired in HD but not PD patients and normal phagocytic capacity was observed in patients after KT as long as renal function was stable. These findings suggest on the one hand, that the dialysis modality is crucial in the development of neutrophil dysfunction and on the other hand that neutrophil dysfunction is reversible after KT. PD patients have a shorter dialysis vintage and a higher residual diuresis in our study. Others have also shown that uraemia and the dialysis modality affect neutrophil phagocytosis and that impaired phagocytic capacity is reversible^30^,^31^. We could also link neutrophil phagocytosis to the risk of infection; a phagocytic capacity below 72% was associated with significantly higher mortality due to infections during follow up.

Since endotoxin is commonly derived from the gut, we also assessed gut permeability in our study cohort. Gut permeability, as measured by differently sized polyethylene glycols, is increased in patients with ESRD^16^. Furthermore bacterial overgrowth has been found in ESRD patients, which might additionally increase gut permeability and bacterial translocation^32^,^33^. ESRD patients often suffer from fluid volume overload, which has been shown to increase gut permeability and correlate with systemic endotoxin levels^34^. Over-hydration has a similar prevalence in both HD and PD^35^. However, in our study we only found significant endotoxemia in HD patients. From our view, over-hydration cannot be the only cause for endotoxemia observed in HD patients. For determination of gut permeability, the current gold standard is the measurement of urinary sugar recovery but this test cannot be performed in ESRD because of decreased eGFR and oftentimes minimal urine output^36^. Therefore, the use of surrogate biomarkers is necessary. We investigated DAO as a serum biomarker of gut permeability and LBP and sCD14 as surrogate parameters for bacterial translocation. DAO is an active intracellular molecule in the cells of the intestinal mucosa and reaches circulation when the barrier function of the gut is impaired. Thus, increased DAO levels represent increased gut permeability. DAO has been used as a biomarker for intestinal barrier dysfunction in various diseases and has been cross validated with other markers of gut permeability^37^,^38^,^39^,^40^,^41^,^42^,^43^.

We found elevated DAO, LBP and sCD14 levels in CKD stage G3–5 patients, dialysis patients and KT patients. No differences were found in DAO, sCD14 and LBP between HD and PD. These findings support the hypothesis that increased gut permeability may be caused by intestinal hypoperfusion due to a reduction in splanchnic blood flow during HD caused by compensatory mechanisms retaining hemodynamic stability during ultrafiltration. We have recently shown that hepato-splanchnic blood flow substantially decreases during HD as a result of an active splanchnic vasoconstriction thus weakening the gut barrier, especially in diabetic subjects^44^. Since we collected the serum samples before the start of HD, our study was not designed to detect bacterial translocation across an ischemic (“stunned”) gut barrier directly. However, our data provide further support for such a relationship as bacterial translocation might predominantly occur in the post-dialytic period^45^. Increased LBP and sCD14 levels in all patient groups might be due to transient or low-grade endotoxemia, which is under the detection limit of 3 EU/ml in the endotoxin assay. Central venous catheters are known sources of endotoxemia too. We did not detect differences in endotoxemia between the different types of vascular access, but the patient numbers for these subgroups were very low.

An altered gut microbiome composition could be a further driving force for endotoxemia, as discussed in a recent review^46^ and could also contribute to the increased risk of infection. Further next-generation sequencing studies with larger patient numbers are necessary to elucidate how different stages of CKD and different renal replacement therapy modalities impact on the composition and function of the gut microbiome in renal insufficiency.

To further investigate the potential mechanism behind neutrophil dysfunction we studied the cellular energy status of isolated neutrophils. This has been associated with decreased neutrophil phagocytic capacity due to TLR4 mediated endotoxin stimulation and exhaustion of cells^47^. Interestingly, we did not find any alterations in energy charge of neutrophils in our patient cohort. Energy status was neither correlated with CKD stage nor with the type of renal replacement therapy. ADP and ATP were significantly reduced in all patient groups compared to controls, which makes a direct relationship to endotoxemia and neutrophil phagocytosis unlikely. Another possible explanation for the observed neutrophil dysfunction might be an interaction of neutrophils with the dialysis membrane, causing an inadequate activation of neutrophils^48^.

Besides the above-described defect in phagocytosis, chronic activation of the innate immune system is highly prevalent in CKD^49^. This leads to increased levels of inflammatory markers (cytokines, acute-phase-proteins)^50^, which are known contributors to the increased mortality in ESRD^51^. Both, oxidative stress and inflammation are highly prevalent in chronic kidney disease patients^52^ and are in close interaction^53^. Neutrophils of patients undergoing HD show an increase in oxidative burst^54^ which contributes to the oxidation of plasma proteins especially albumin^55^. Recently a misbalance in the “gut microbiome - immune system interaction” was proposed to further contribute to systemic inflammation^56^. We measured a panel of cytokines in serum and found that all measured parameters (IL6, sIL6R, IL8, IL10, TNFα, TNFR1 and TNFR2) were elevated in CKD patients compared to controls. We found no clear association with the degree of renal impairment. In HD patients the anti-inflammatory cytokine IL10 was lower in serum compared to PD, whereas TNFR1 and TNFR2 were nearly doubled.

**Figure 4:**
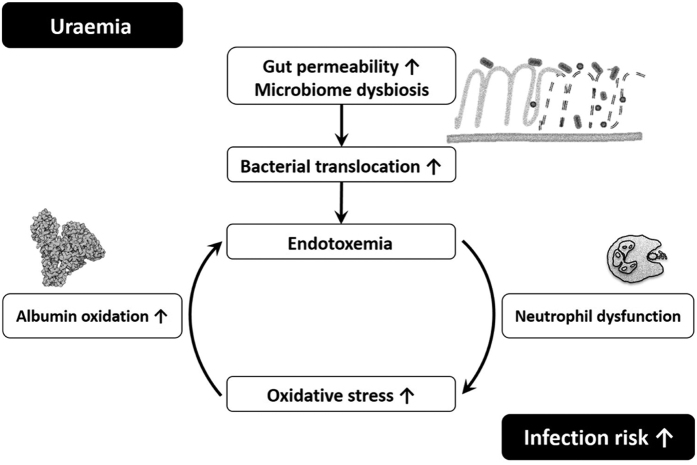
The hypothesis of a vicious cycle in chronic renal disease. The risk of infection increases as result of a disturbed gut barrier and endotoxemia leading to neutrophil dysfunction.

Neutrophils of patients undergoing HD had a slightly, but significantly higher resting oxidative burst but their ability to increase burst through a mild or high stimulus was worse than in PD patients. We could also confirm previously published results on oxidized proteins (AOPPs, albumin) and carbonylated proteins, which were significantly elevated in all patient groups and also showed a correlation with the stage of renal insufficiency (oxidized albumin). AOPPs and CP were also significantly higher in HD versus PD patients. Oxidized albumin^57^ and AOPPs^58^ are not only the result of neutrophil oxidative burst damage^59^ but can in turn again activate neutrophils. This possibly creates a vicious cycle leading to additional neutrophil dysfunction, which in turn increases the risk of infections (Fig. 4).

The limitation of our study is the cross sectional, observational nature of the study which does not allow proving causality. However, the relatively large number of patients of a broad CKD-spectrum strengthens the results of this study. Another limitation is the fact, that the current gold standard for measuring intestinal permeability cannot be performed in ESRD patients, as described above. We however used different biomarkers for intestinal permeability and bacterial translocation that have been described in literature. Since we could not detect a correlation of these markers (DAO, sCD14, LBP) with renal function, we are confident that these markers reflect intestinal permeability in our patient cohort. Furthermore, measurement of endotoxin in serum samples is difficult. None of the commercially available LPS detection kits are recommended to be used for human serum samples. This paradox has been discussed before, but the Limulus Amoebocyte Assay is still in use, lacking valid alternatives^60^,^61^. The main problem is the presence of many inhibiting substances in serum, that can influence any of the enzymes of the Limulus Amoebocyte Assay cascade leading to decreased or false negative results^62^,^63^. We therefore adapted the HEK cell based assay from Invitrogen for human serum use. This assay is based on the ability of TLR4 to recognize structurally different LPS from gram-negative bacteria. To our knowledge, we are the first to describe this assay in the context of renal insufficiency. It has been previously shown that serum from uremic patients causes a down-regulation of TLR4 expression on monocytes. Therefore, we do not assume a false positive TLR4 signalling by patient sera. Additionally, cells were stimulated by serum samples in the presence of detection medium which reduced the concentration of of uremic toxins thereby minimizing the effect on HEK-cells. The absolute endotoxin levels resulting from the HEK cell based assay are significantly higher than endotoxin levels reported from the Limulus Amoebocyte Assay. In another study from our group in patients with liver cirrhosis we also observed higher endotoxin levels in relatively stable patients^64^. At the moment we can only speculate on the reasons. One reason might be that we used a standard curve in serum from healthy controls for this assay, whereas most users of the Limulus Amoebocyte Assay use a standard curve in water. Due to various inhibiting substances in serum, this might lead to lower absolute endotoxin levels in the Limulus Amoebocyte assay.

In summary, although endotoxemia, increased gut permeability, oxidative stress and systemic inflammation were present in all CKD patients, endotoxemia was associated with impaired neutrophil function only in HD patients. Neutrophil energy status did also not explain the differences in neutrophil function. HD patients in our study cohort not only had increased endotoxemia and impaired neutrophil function but also died more often from infections. Therefore, further studies are needed to explore the reason for endotoxemia and neutrophil dysfunction especially in HD patients. Interventions to decrease the risk for infections in ESRD are an unmet clinical need and further investigations in this field will make targeted interventions possible.

## Methods

We enrolled CKD patients with stages G3 to 5, patients with ESRD undergoing dialysis treatment and patients after KT at the Department of Internal Medicine, Clinical Division of Nephrology, Medical University of Graz. The Ethics Committee of the Medical University Graz (IRB00002556) approved the study protocols (23–056 ex10/11, NCT01362569). The study was conducted according to the Declaration of Helsinki and all participants gave written informed consent prior enrolment. Patients with malignancy, pregnancy, chronic inflammatory bowel disease, celiac disease, active alcohol abuse, severe organ dysfunction unrelated to renal dysfunction or patients with clinical evidence of active infection were excluded. Age and sex matched healthy controls were included in the study. These subjects had no evidence of renal disease and did not take any medication. Patients were followed-up and survival data were censored upon December 1, 2015. Causes of death were stratified into infectious causes, non-infectious causes and unknown reasons.

Blood samples were taken with sterile equipment (Vacuette, Greiner, Austria) and serum was stored in Eppendorf safe-lock tubes, 1.5 ml (Eppendorf, Vienna, Austria). Equipment was tested for endotoxin and no endotoxin was detected. Routine laboratory parameters were analysed at the Clinical Institute of Medical and Chemical Laboratory Diagnostics of the Medical University of Graz. The estimated glomerular filtration rate (eGFR) was calculated according to CKD-EPI creatinine equation (2009).

The Phagotest^®^ (Glycotope, Heidelberg, Germany) was used as directed by the manufacturer to measure phagocytosis by flow cytometric analysis using FITC-labelled opsonized *E. coli* bacteria. In brief: heparinized whole blood (100 μl) was incubated with 20 μl of FITC-labelled opsonized *E. coli* bacteria for 10 min either on ice (control) or at 37 °C. Fluorescence of surplus bacteria was quenched by addition of 100 μl of quenching solution. After 2 washing steps (3 ml wash solution) erythrocytes were lysed by addition of lysing solution (2 ml) and incubated for 20 min at room temperature. Ten minutes prior to flow cytometric measurement DNA staining solution (200 μl) was added to each sample. A forward-side scatter gate was set on neutrophils and 10,000 neutrophils were recorded. Percentage of neutrophils that showed no phagocytic activity was recorded (inactive neutrophils). The phagocytic capacity was calculated by weighing the geometric mean of fluorescence intensity (GMFI) with the percentage of low and high phagocytizing neutrophils. To overcome batch variations all values are presented as *n*-% change of healthy controls determined by the corresponding batch of bacteria. An intra-assay precision for percentage of phagocytizing cells and GMFI is given with 0.2% CV and 1.5% CV by the supplier. Quality control was carried out daily by using CS&T beads.

The Phagoburst^®^ kit (Glycotope, Heidelberg, Germany) was used as directed by the manufacturer to determine the percentage of neutrophils that produced reactive oxidants by bacterial (*E. coli*) stimulation, low stimulation (fMLP - priming) or without stimulation analysed by flow cytometry. In brief: heparinized whole blood (100 μl) was incubated with washing solution (resting burst), fMLP (N-formyl-Met-Leu-Phe; priming) or opsonized *E. coli* bacteria (bursttest) (20 μl each) for 10 min at 37 °C. Afterwards substrate solution (20 μl) were added to each tube and incubated for 10 min at 37 °C. Erythrocytes were then lysed by addition of lysing solution (2 ml) and incubated for 20 min at room temperature. Ten minutes prior to flow cytometric measurement DNA staining solution (200 μl) was added to each sample. A forward-side scatter gate was set on neutrophils and 10,000 neutrophils were recorded. Data are presented as percentage of bursting neutrophils (FITC positive). Background signal (bursting neutrophils without stimulus) was subtracted from stimulated bursting neutrophils. An intra-assay precision for percentage of oxidizing cells is given with 0.1% CV by the supplier. Flow cytometric analysis was done with an LSRII cytometer in combination with FACS Diva 6.2 software (BD Bioscience, Heidelberg, Germany). Quality control was carried out daily by using CS&T beads.

Cellular energy status in neutrophils was measured by HPLC as described in^65^,^66^,^67^. In brief: neutrophils were isolated by layering whole blood (5 ml) over Polymorphprep^TM^ (5 ml) (Axis-Shield, Oslo, Norway) and centrifuged at 500 g for 35 min at room temperature. Residual erythrocytes within the neutrophil phase were lysed by addition of Red Cell Lysis Buffer (2 ml) (Roche Diagnostics, Vienna Austria). Neutrophils were suspended in HBSS to the desired concentration. Neutrophils (10^6^) were deproteinized with 0.4 mol/l perchloric acid (200 μl). After centrifugation the acid extract (200 μl) was neutralized with of 2 mol/l potassium carbonate (~15 μl, 4 °C). The supernatant obtained after centrifugation was used for HPLC analysis (injection volume: 40 μl). The pellet of the acid extract was dissolved in 0.1 mol/l sodium hydroxide (250 μl) for protein determination (BCA Protein Assay, Pierce). The HPLC analytical method for separation of the high energy phosphates has been reported previously^65^,^66^,^67^. Procedure in brief: Separation is performed on a Hypersil ODS column (5 μm, 250 mm × 4 mm I.D.) using a L-2200 autosampler, two L-2130 HTA pumps and a L-2450 diode array detector (all: VWR Hitachi, Austria). Detector signals (absorbance at 214 nm and 254 nm) were recorded and analysed by EZchrom Elite software (Agilent Technologies, Santa Clara, USA).

Energy charge was calculated by following formula: EC  =  (ATP + ½ ADP)/(AMP + ADP + ATP). AMP, ADP and ATP concentrations were represented as μmol/l.

The advanced oxidized protein products (AOPP) - assay was performed as described previously^68^,^69^. In brief, serum was depleted of ApoB-containing lipoproteins with polyetylenglycol (PEG). Precipitate was pelleted by centrifugation (10.000 × g, 30 min) and the supernatant was used for AOPP detection. Subsequently, apoB-depleted serum was mixed 0.2 mol/l citrate buffer and incubated for 2 min on a shaker. Absorbance was measured on a Nano Drop 1000 (Peqlab, Germany) spectrophotometer at 340 nm. AOPP concentrations were calculated by a chloramine-T standard curve ranging from 1 to 100 μmol/l. Carbonyl content of proteins was measured by ELISA (Immundiagnostik, Erlangen Germany) according to the manufacturers’ instructions. The redox state of albumin was analysed in serum as described previously^70^. Samples were diluted 1:100 in sample buffer (0.1 mol/l sodium phosphate, 0.3 mol/l sodium chloride, pH 6.87) and filtered with a 0.45 μm nylon membrane (Whatman International Ltd, Maidstonem, UK). Filtrate (25 μl) of was injected and separated with an anion exchange column (Shodex Asahipak ES-502 N7C, Showa Denko, Munich, Germany) using 50 mmol/l sodium acetate, 400 mmol/l sodium sulfate, pH 4.85 as mobile phase. A gradient pump (FLUX Rheos 4000; Spectronex GmbH, Austria) applied a flow rate of 1 ml/min with an ethanol gradient reaching from 0 to 6%. The column was kept at a temperature of 35 °C. Fluorescence emission was detected by 280/340 nm with a Jasco 821FP detector (Spectronex GmbH, Vienna, Austria). Fraction quantification was done by comparing peak heights by means of EZChrom Elite chromatography software (Agilent Technologies, Santa Clara, USA) and expressed as the percent of total albumin. Thereby, reversibly oxidized albumin (human nonmercapt-albumin 1, HNA1) and irreversibly oxidized albumin (human nonmercapt-albumin 2, HNA2) were quantified.

An adapted Human embryonic kidney cells (HEK)-blue LPS detection kit (InvivoGen, San Diego, USA) was used to determine serum endotoxin levels via a cell based colorimetric assay. Endotoxin free equipment (endotoxin free glass test tubes, LAL reagent endotoxin free water (Charles Rivers, Massachusetts, USA), 6-well plates with endotoxin levels ≤0,1 EU/ml (Corning, New York, USA), low-endotoxin fetal bovine serum (Gibco, Vienna, Austria), ART Barrier tips (Fisher scientific, Vienna, Austria), combitips advanced (Biopur, Eppendorf, Vienna Austria) was used. Briefly, HEK-cells were subcultured when they reached a confluency of 80%. Cells (5×10^4^/well were incubated in 2 ml complete growth media (DMEM high glucose supplemented with 1% Pen/Strep, 10% FBS, 1× Normocin, 1× Selection mix) at 37 °C, 5% CO_2_ in a 24-well plate overnight. A five-point endotoxin standard curve (50 EU/ml to 3.125 EU/ml prepared in serum of a healthy donor) and positive controls were prepared. Growth medium was changed to detection medium (950 μl). Samples, standards and controls were added in triplicates (50 μl) and incubated overnight. Each sample was corrected for its specific alkaline phosphatase activity to avoid overestimation of endotoxin concentration using a regression model. On the next day supernatant (100 μl) was transferred in duplicates to a 96-well plate and quantified photometrically by reading the OD at 650 nm.

Soluble (s)CD14 and lipopolysaccharide binding protein (LBP) were both measured by “sandwich” ELISA (Hycult, Netherlands). A ready-to-use solid-phase “sandwich” ELISA (Immundiagnostik AG, Bensheim, Germany) was used to detect diamine-oxidase (DAO) in serum samples. All ELISA Kits have been performed exactly as suggested by the manufacturer.

Interleukins (IL6, IL8, IL10) as well as tumour necrosis factor- α (TNF-α), and soluble TNF receptor (sTNFR) -1 were analysed by FlowCytomix (eBioscience, Vienna Austria). Both, soluble IL6-receptor (sIL6R) and sTNFR2 were determined by “sandwich” ELISA (eBioscience, Vienna, Austria). All Kits have been performed exactly as suggested by the manufacturer.

### Statistics

All statistical analyses were performed using SPSS version 23.0 (SPSS Inc., Chicago, IL) and GraphPad Prism version 5.0 for visualisations. Comparison of two independent groups with normal distributed parameters was tested for homogeneity (Levene’s test) and accordingly assessed by T-test or Welch; three or more groups were compared by ANOVA using Tukey’s or Games-Howell’s correction. For parameters, which were not normally distributed, non-parametric tests were used (Mann Whitney U test, or Kruskal-Wallis and Wilcoxon signed rank test). Two-tailed Pearson or Spearman tests were used for metrical data correlation. Nominal and ordinal scaled data comparison was assessed by Chi-square test using Pearson or Fisher’s Exact test. Survival analysis were performed by Kaplan Meier curves and assessed by Log-Rank (Mantel-Cox). ROC curve and Youden index were used to define a cut-off value. All statistical tests were 2-sided, and p-values < 0.05 were considered statistically significant. Data are presented as median and quartiles (Q_1_, Q_3_).

## Additional Information

**How to cite this article**: Lemesch, S. *et al*. Mode of renal replacement therapy determines endotoxemia and neutrophil dysfunction in chronic kidney disease. *Sci. Rep.* **6**, 34534; doi: 10.1038/srep34534 (2016).

## Figures and Tables

**Table 1:** Patient characteristics.

	CKD stage 3–5			Dialysis (HD + PD)			Dialysis subgroups						KT			Healthy controls		
	n = 57			n = 60			HD n = 32			PD n = 28			n = 67			n = 25		
Age [years]	61	(50, 68)		56	(42, 68)		56	(44, 70)		54	(35, 66)		56	(47, 66)		50	(44, 59)	
Sex [male], % (n)	63	(36)		62	(37)		53	(17)		71	(20)		63	(42)		48	(12)	
BMI [kg/m^2^]	27.7	(24.7, 34.8)		26.3	(23.4, 30.4)		24,6	(22.6, 30.1)		27, 8	(24, 30.4)		25.3	(23.0, 28.6)	c	26,7	(24.5, 27.8)	
Dialysis vintage [months]		—		41	(21, 89)		70	(27, 154)	§	29	(18, 44)		61	(12, 138)		—		
Residual diuresis [ml]				850	(0, 3800)		50	(0, 563)	§	1225	(638, 1850)							
**Medication % (n)**																		
Sevelamer	4	(2)		52	(31)		50	(16)		54	(15)		—				—	
Immunosuppression	11	(6)		5	(3)		9	(3)		0	(0)		100	(67)			—	
**Aetiology of renal disease % (n)**																		
Immunological‡	32	(18)		27	(16)		19	(6)		36	(10)		45	(30)			—	
Non-immunological‡	63	(36)		40	(24)		44	(14)		36	(10)		31	(21)			—	
other	5	(3)		33	(20)		38	(12)		29	(8)		24	(16)			—	
**Comorbidities**																		
Charlson Comorbidity Index	5	(2, 9)		4	(2, 12)		4	(2, 12)		4	(2, 12)		4	(2, 10)			—	
Cardiovascular diseases, % (n)	69	(39)		50	(30)		59	(19)		39	(11)		64	(43)			—	
Diabetes, % (n)	35	(20)		28	(17)		38	(12)		18	(5)		25	(17)			—	
**Laboratory parameters**																		
eGFR [ml/min/1,73 m^2^]	23.2	(16.0, 31.1)	a	5.7	(4.8, 8.0)	b	5,8	(5.0, 7.4)	*	5,7	(4.5, 8.9)	*	43.5	(31.0, 58.0)	c	82.0	(61.2, 93.7)	**
Albumin [g/dl]	4.2	(3.9, 4.4)	a	4.0	(3.7, 4.2)	b	4	(3.7, 4.3)	*	3,9	(3.7, 4.1)	*	4.4	(4.2, 4.6)	c	4.7	(4.6, 5.0)	**
Creatinine [mg/dl]	2.5	(2.0, 3.6)	a	8.1	(6.7, 10.0)	b	8	(6.7, 9.8)	*	8,4	(6.6, 11.4)	*	1.6	(1.2, 2.0)	c	0.9	(0.8, 1.0)	**
Urea [mg/dl]	93	(66, 131)		98	(84, 113)	b	98	(77, 109)	*	99	(89, 125)	*	59	(47, 73)	c	32	(27, 36)	**
Phosphate [mmol/l]	1.2	(1.0, 1.3)	a	1.6	(1.3, 1.9)	*, b	1,7	(1.2, 2.0)	*	1,5	(1.3, 1.9)	*	0.9	(0.8, 1.1)	c	1.0	(0.8, 1.1)	
CRP [mg/l]	2.4	(1.3, 6.5)		3.0	(0.9, 9.1)		7.4	(1.0, 13.6)	*, §	1.8	(0.8, 4.1)		2.3	(0.9, 4.8)		1.6	(0.6, 2.4)	
Neutrophil count [G/l]	4.2	(3.5, 4.5)		4.4	(3.3, 6.0)		4	(3.1, 6.0)		4,4	(3.5, 5.7)		4.2	(3.2, 5.7)		4.2	(3.3, 4.9)	
Neutrophil/lymphocyte ratio	3.1	(2.1, 4.1)		2.9	(1.9, 4.5)		3,2	(2.0, 5.4)	*	2,6	(1.8, 4.0)	*	3.3	(2.2, 4.7)		1.8	(1.5, 2.4)	**
**Deceased % (n)**																		
All-cause mortality	9	(5)	a	32	(19)	b	38	(12)		25	(7)		8	(5)			—	
Infectious-cause mortality	1.8	(1)	a	15	(9)	b	25	(8)	§	3.6	(1)		5	(3)			—	

CKD chronic kidney disease, HD haemodialysis, PD peritoneal dialysis, KT after kidney transplantation, BMI Body mass index, eGFR estimated glomerular filtration rate, CRP C-reactive protein.

a, b, c significant difference to another patient group (a: CKD to Dialysis, b: Dialysis to KT, c: KT to CKD), ^*^significant difference to healthy controls, ^**^all patient groups (CKD, Dialysis, KT) differ significantly from healthy controls, **Dialysis subgroups:** ^§^significant difference between HD and PD patients, ^*^significant difference to healthy controls.

-data not available, data is shown as median (Q1, Q3) unless stated otherwise.

^‡^Immunological: glomerulonephritis, Non-immunological: cystic kidneys, diabetic-/ vascular nephropathy.

**Table 2:** Endotoxin, gut permeability, neutrophil function oxidative stress and inflammation in ESRD.

	CKD stage 3–5			Dialysis			Dialysis subgroups						KT			Healthy controls		
	n = 57			n = 60			HD n = 32			PD n = 28			n = 67			n = 25		
**Endotoxemia and gut permeability**																		
Endotoxin [EU/ml]	1.3	(0.0, 5.7)		5.4	(0.0, 13.3)	*	8.9	(0.0, 15.6)	*, §	0	(0.0, 7.0)		1.0	(0.0, 7.0)		0.0	(0.0, 1.8)	
LBP [μg/ml]	34.8	(25.7, 44.0)		28.3	(21.4, 40.5)		28.3	(22.7, 49.0)	*	28.3	(18.6, 37.3)	*	27.0	(19.7, 37.8)		14.3	(12.7, 20.6)	**
sCD14 [μg/ml]	2.7	(2.2, 4.1)		2.9	(2.5, 3.9)	b	3.3	(2.6, 4.2)	*	2.8	(2.5, 3.2)	*	2.3	(1.9, 2.8)		1.7	(1.3, 2.3)	**
DAO [ng/ml]	33.1	(26.3, 44.4)		26.3	(20.6, 37.5)		31.9	(21.9, 44.8)	*	25.6	(20.0, 30.0)		31.9	(22.5, 39.4)		19.7	(14.5, 34.4)	**
**Neutrophil function**																		
Phagocytosis [% change to healthy controls]	103.9	(83.6, 166.5)	a	76.2	(61.0, 101.2)	*, b	70.6	(49.6, 87.1)	*, §	92.2	(65.9, 135.3)		100.8	(76.9, 152.1)		100.0	(83.5, 129.2)	
Inactive neutrophils [%]	1.60	(1.0, 4.9)		2.6	(1.5, 5.9)	*	2.6	(1.4, 4.3)	*	1.8	(1.2, 5.5)	*	2.2	(1.4, 4.4)	*	1.1	(0.5, 1.6)	
Energy charge	0.79	(0.75, 0.84)		0.81	(0.77, 0.85)		0.80	(0.76, 0.84)		0.82	(0.78, 0.88)		0.81	(0.77, 0.86)		0.82	(0.77, 0.85)	
Resting burst (no stimulus) [%]	3.1	(2.4, 4.4)		3.1	(2.5, 3.9)		3.6	(2.7, 4.6)	*, §	2.8	(2.3, 3.4)		3.7	(2.7, 5.4)	*	2.8	(2.3, 3.2)	
Priming (low stimulus by fMLP) [%]	1.4	(0.0, 5.4)		3.3	(1.0, 6.0)		1.6	(0.6, 3.5)	§	4.2	(2.9, 7.3)		2.9	(1.0, 6.0)		2.7	(1.5, 4.6)	
Burst (bacterial stimulus by *E. coli*) [%]	93.7	(90.7, 95.7)		94.1	(89.6, 95.4)		90.9	(85.7, 94.6)	*, §	95	(93.9, 95.9)	*	92.6	(86.6, 95.6)		96.2	(95.4, 97.0)	**
**Oxidative stress**																		
AOPP [μmol/l]	54.9	(33.5, 74.1)	a	73.6	(55.5, 98.6)	b	91	(63, 114)	*, §	67	(51, 80)	*	53.5	(35.2,70.2)		30.7	(26.4, 36.8)	**
HMA [%]	57.4	(52.6, 63.9)		61.3	(56.4, 67.8)		59.1	(54.1, 67.8)	*	64.3	(60.2, 67.9)	*	63.3	(59.4, 67.8)	c	73.6	(69.6, 76.3)	**
HNA1 [%]	40	(33.4, 44.7)	a	35.2	(28.7, 40.2)		38.0	(28.7, 42.6)	*	31.9	(28.6, 37.1)	*	33.5	(29.8, 37.2)	c	25.0	(21.4, 28.0)	**
HNA2 [%]	2.1	(1.9, 2.7)	a	3.3	(2.8, 3.9)	*	3.2	(2.8, 4.0)	*	3.4	(2.7, 3.9)	*	2.9	(2.3, 3.8)	*	2.4	(1.8, 2.8)	
CP [pmol/mg protein]	206.7	(190.4, 223.0)		199.3	(173.3, 223.9)		207	(182, 235)	*, §	183	(170, 208)		206.9	(186.1, 224.4)	c	175.1	(143.0, 194.0)	**
**Inflammation**																		
Ferritin [ng/ml]	126	(56, 219)	a	237	(128, 433)	b	349	(222, 519)	*,§	163	(94, 234)		126	(68, 254)		135	(71, 286)	
Transferrin saturation [%]	23	(18, 33)		26	(20, 31)		27	(19, 32)		25	(20, 30)		26	(20, 33)		28	(22, 38)	
**Cytokine [pg/ml] and -receptor [ng/ml] panel**																		
IL6	2.0	(0.9, 3.7)		3.8	(2.1, 6.8)	b	4.5	(1.8, 9.9)	*	3.0	(2.1, 4.8)	*	1.8	(0.5, 3.3)		0.0	(0.0, 0.1)	**
sIL6R	250	(225, 325)	*, a	225	(175, 275)	*	225	(175, 275)		225	(181, 275)	*	225	(175, 275)	c	185	(155, 225)	
IL8	15.2	(6.8, 25.7)		10.8	(5.7, 20.1)		13.7	(5.5, 26.0)	*	10.6	(7.3, 12.4)	*	13.4	(7.2, 20.6)		1.3	(0.0, 2.4)	**
IL10	2.1	(0.3, 4.7)		3.4	(1.5, 7.6)		2.5	(0.0, 5.0)	*, §	6.1	(2.4, 10.6)	*	1.9	(0.0, 5.6)		0.0	(0.0, 0.0)	**
TNFα	0.9	(0.0, 5.5)		1.0	(0.0, 5.0)		0.0	(0.0, 4.6)		2.0	(0.0, 5.5)	*	0.0	(0.0, 5.7)		0.0	(0.0, 0.0)	**
sTNFR1	7.9	(5.2, 11.7)	a	25.1	(16.4, 33.7)	b	28.9	(22.4, 39.6)	*	18.3	(12.4, 27.8)	*	4.5	(2.6, 6.2)	c	1.0	(0.7, 1.3)	**
sTNFR2	14	(11, 18)		19	(11, 25)	*, b	24	(20, 29)	*, §	11	(8, 16)		10	(7, 15)		5	(4, 22)	

CKD chronic kidney disease, HD haemodialysis, PD peritoneal dialysis, KT kidney transplantation, LBP LPS binding protein, sCD14 soluble CD14, DAO diamine oxidase, AOPP advanced oxidation protein products, HMA human mercaptalbumin, HNA human non-mercaptalbumin, CP carbonylated proteins, IL interleukin, sIL6R soluble interleukin 6 receptor, TNFα Tumour necrosis factor α, sTNFR1/2 soluble TNF receptor ½.

a, b, c significant difference to another patient group (a: CKD to Dialysis, b: Dialysis to KT, c: KT to CKD), ^*^significant difference to healthy controls, ^**^all patient groups (CKD, Dialysis, KT) differ significantly from healthy controls, **Dialysis subgroups:** ^§^significant difference between HD and PD patients, ^*^significant difference to healthy controls, -data not available, data is shown as median (Q1, Q3) unless stated otherwise.

**Table 3:** Influence of vascular access in patients undergoing haemodialysis.

	Types of vascular access								
	AVF		p	AVG		p	CAT		p
Vascular access % (n)	53	(17)		19	(6)		28	(9)	n.s.
endotoxin [EU/ml]	7.4	(0.0, 13.6)		8.9	(0.0, 15.2)		10.8	(3.5, 27.8)	n.s.
sCD14 [μg/ml]	3.4	(2.5, 4.9)		2.9	(2.5, 3.6)		3.6	(2,8, 4.2)	n.s.
LBP [μg/ml]	28.2	(21.1, 52.4)		45.5	(20.4, 65.4)		26.6	(23.3, 36.1)	n.s.
DAO [ng/ml]	28.8	(19.4, 38.1)		26.3	(24.2, 47.0)		36.9	(26.9, 55.0)	n.s.
phagocytosis [% change to healthy controls]	61.6	(46.9, 92.7)		87.2	(72.7, 111.9)		87.2	(72.7, 111.9)	n.s.
TNFα [pg/ml]	0.7	(0.0, 5.4)		3.6	(0.4, 13.5)	§	0.0	(0.0, 0.0)	
sIL6R [ng/ml]	225	(175, 263)		162	(150, 238)	§	350	(193, 413)	
sTNFR1 [ng/ml]	28.0	(22.6, 31.7)		21.6	(13.5, 29.3)	§	44.1	(33.0, 45.6)	
CRP [mg/l]	1.6	(0.6, 9.7)	§	8.0	(1.3, 25.2)		9.5	(8.1, 36.0)	
**Mortality % (n)**									
All causes	34	(4)		33	(2)		67	(6)	n.s.
Infections	24	(4)		33	(2)		22	(2)	n.s.

^§^Significant difference to patients with catheter, n.s. not significant, Median (Q_1_, Q_3_) unless stated otherwise.

AVF arteriovenous fistula, AVG arteriovenous graft, CAT catheter, sCD14 soluble CD14, LBP LPS binding protein, DAO diamine-oxidase, sIL6R soluble interleukin 6 receptor, sTNFR1 soluble TNF receptor 1.

**Table 4:** Chronic kidney disease patients with different stages of renal insufficiency (eGFR).

	eGFR [ml/min/1,73 m^2^]								
	30–59		p	15–29		p	<15		p
	n = 17			n = 29			n = 11		
Age [years]	55	(41, 62)		62	(49, 71)		64	(53, 72)	c
Sex [male], % (n)	71	(12)		55	(16)		72	(8)	
BMI [kg/m^2^]	26.1	(23.9, 30.5)		30.5	(24,8, 37.5)		27.5	(24.7, 31.7)	
**Medication % (n)**									
Sevelamer	0	(0)		3	(1)		9	(1)	
Immunosuppression	18	(3)		10	(3)		0	(0)	
**Aetiology of renal disease % (n)**									
Immunological ‡	41	(7)		28	(8)		27	(3)	
Non-immunological ‡	59	(10)		64	(18)		72	(8)	
other	0	(0)		10	(3)		0	(0)	
**Comorbidities % (n)**									
Charlson Comorbidity Index	0	(0, 3)	a	4	(0, 5)		0	(0, 4)	
Cardiovascular diseases	59	(10)		66	(19)		91	(10)	
Diabetes	24	(4)		45	(13)		27	(3)	
**Laboratory parameters**									
eGFR [ml/min/1,73 m^2^]	41.6	(33.3, 52.2)		21.2	(18.5, 26.4)		13.0	(9.5, 14.2)	**
Albumin [g/dl]	4.2	(3.8, 4.4)		4.2	(4.0, 4.4)		4.1	(3.9, 4.2)	
Creatinine [mg/dl]	1.7	(1.4, 2.1)		2.6	(2.2, 3.4)		4.7	(3.7, 5.4)	**
Urea [mg/dl]	57	(47, 80)		96	(82, 128)		144	(120, 153)	**
Phosphate [mmol/l]	1.1	(0.9, 1.3)		1.1	(0.9, 1.2)	b	1.5	(1.2, 1.8)	c
CRP [mg/l]	1.7	(0.6, 6.2)		2.4	(1.4, 6.3)		5.0	(1.6, 13.9)	
Ferritin [ng/ml]	150	(76, 229)		83	(47, 181)		184	(90, 222)	
Transferrin saturation [%]	29.0	(19, 34)		22.0	(19, 32)		21.0	(15, 32)	
Neutrophil count [G/l]	4.1	(3.2, 5.5)		4.8	(3.8, 5.5)		3.9	(3.7, 4.6)	
Neutrophil/lymphocyte ratio	2.7	(2.1, 3.6)		3.3	(2.0, 4.1)		3.2	(2.4, 5.4)	
**Endotoxemia and gut permeability**									
Endotoxin [EU/ml]	1.4	(0.0, 4.9)		0.0	(0.0, 7.4)		3.0	(0.0, 6.2)	
LBP [μg/ml]	24.8	(17.6, 36.7)		34.8	(29.8, 45.2)		38.1	(26.6, 48.8)	
sCD14 [μg/ml]	2.2	(1.9, 3.2)		3.0	(2.2, 5.5)		2.5	(2.3, 3.4)	
DAO [ng/ml]	39.4	(27.5, 49.7)		31.9	(24.2, 41.3)		32.5	(26.7, 45.3)	
**Neutrophil function**									
Phagocytosis [% change to healthy controls]	134.3	(98.3, 189.3)		92.6	(83.1, 125.0)		107.3	(63.0, 167.3)	
Inactive neutrophils [%]	1.9	(0.8, 7.2)		1.5	(1.1, 5.4)		1.6	(0.9, 3.6)	
Energy charge	0.84	(0.77, 0.87)		0.77	(0.74, 0.83)		0.79	(0.76. 0.87)	
Resting burst (no stimulus) [%]	2.4	(1.8, 2.9)	a	3.4	(1.8, 2.9)		4.1	(3.1, 5.2)	c
Priming (low stimulus by fMLP) [%]	0.7	(0.0, 4.7)		2.7	(0.7, 6.6)		0.1	(0.0, 3.6)	
Burst (bacterial stimulus by *E. coli*) [%]	94.4	(90.7, 96.1)		94.4	(92.4, 95.9)		92.0	(88.7, 93.4)	
**Oxidative stress**									
AOPP [μmol/l]	43.7	(31.8, 57.3)		57.3	(32.7, 73.1)		73.8	(51.6, 107.5)	
HMA [%]	65.1	(60.1, 67.7)	a	56.5	(51.7, 60.7)		52.1	(47.8, 56.0)	c
HNA1 [%]	33.2	(30.7, 38.2)	a	41.7	(37.5, 45.9)		44.7	(38.7, 48.2)	c
HNA2 [%]	2.0	(1.5, 2.2)		2.1	(1.9, 2.5)	b	3.0	(2.9, 4.1)	c
CP [pmol/mg protein]	197.9	(186.9, 220.8)		204.6	(183.0, 219.2)		214.2	(198.1, 229.3)	
**Cytokine [pg/ml] and -receptor [ng/ml] panel**									
IL6	1.6	(0.6, 2.3)		2.1	(0.8, 3.5)		2.4	(1.3, 8.8)	
sIL6 R	300	(238, 325)		250	(225, 338)		250	(200, 250)	
IL8	12.1	(4.9, 25.1)		9.9	(5.6, 21.0)	b	27.9	(15.2, 58.9)	c
IL10	0.8	(0.0, 4.8)		3.0	(1.2, 4.6)		2.1	(0.0, 5.9)	
TNFα	1.4	(0.0, 5.7)		2.0	(0.0, 5.7)		0.0	(0.0, 4.4)	
sTNFR1	4.3	(2.4, 6.8)		8.4	(6.2, 11.7)		12.6	(10.3, 16.4)	**
sTNFR2	10	(8, 21)		13	(11, 17)		18	(12, 19)	

a, b, c significant difference to another patient group (a: eGFR 30–59 to eGFR 15–29, b: eGFR 15–29 to eGFR <15, c: eGFR <15 to eGFR 30–59), ^**^all patient groups differ significantly from each other, - data not available, data is shown as median (Q1, Q3) unless stated otherwise.

LBP LPS binding protein, sCD14 soluble CD14, DAO Diamine-Oxidase, AOPP Advance Oxidation Protein Products, HMA human mercaptalbumin, HNA human non-mercaptalbumin, CP Carbonylated proteins, IL Interleukin, sIL6R soluble Interleukin 6 receptor, TNFα Tumor necrosis factor α, sTNFR1/2 soluble TNF receptor 1/2.

^‡^Immunological: glomerulonephritis, Non-immunological: cystic kidneys, diabetic-/ vascular nephropathy.

## References

[b1] MacGregor M. S. How common is early chronic kidney disease? A background paper prepared for the UK Consensus Conference on early chronic kidney disease. Nephrology, dialysis, transplantation: official publication of the European Dialysis and Transplant Association - European Renal Association 22 Suppl 9, ix8–18, 10.1093/ndt/gfm444 (2007). 17998238

[b2] Foley R. N., Guo H., Snyder J. J., Gilbertson D. T. & Collins A. J. Septicemia in the United States dialysis population, 1991 to 1999. Journal of the American Society of Nephrology: JASN 15, 1038–1045 (2004). 15034107 10.1097/01.asn.0000119144.95922.c4

[b3] Schmidt T. . CD66b Overexpression and Loss of C5a Receptors as Surface Markers for Staphylococcus aureus-Induced Neutrophil Dysfunction. PloS one 10, e0132703, 10.1371/journal.pone.0132703 (2015). 26176669 PMC4503562

[b4] Kessler M., Hoen B., Mayeux D., Hestin D. & Fontenaille C. Bacteremia in patients on chronic hemodialysis. A multicenter prospective survey. Nephron 64, 95–100 (1993). 8502343 10.1159/000187285

[b5] Powe N. R., Jaar B., Furth S. L., Hermann J. & Briggs W. Septicemia in dialysis patients: incidence, risk factors, and prognosis. Kidney international 55, 1081–1090, 10.1046/j.1523-1755.1999.0550031081.x (1999). 10027947

[b6] Sarnak M. J. & Jaber B. L. Mortality caused by sepsis in patients with end-stage renal disease compared with the general population. Kidney international 58, 1758–1764, 10.1111/j.1523-1755.2000.00337.x (2000). 11012910

[b7] Dhainaut J. F., Claessens Y. E., Janes J. & Nelson D. R. Underlying disorders and their impact on the host response to infection. Clinical infectious diseases: an official publication of the Infectious Diseases Society of America 41 Suppl 7, S481–S489, 10.1086/432001 (2005). 16237651

[b8] Chonchol M. Neutrophil dysfunction and infection risk in end-stage renal disease. Seminars in dialysis 19, 291–296, 10.1111/j.1525-139X.2006.00175.x (2006). 16893406

[b9] Cohen G., Raupachova J. & Horl W. H. The uraemic toxin phenylacetic acid contributes to inflammation by priming polymorphonuclear leucocytes. Nephrology, dialysis, transplantation: official publication of the European Dialysis and Transplant Association - European Renal Association 28, 421–429, 10.1093/ndt/gfs454 (2013). 23229930

[b10] Haag-Weber M. & Horl W. H. Dysfunction of polymorphonuclear leukocytes in uremia. Seminars in nephrology 16, 192–201 (1996). 8734462

[b11] Mera K. . Oxidation and carboxy methyl lysine-modification of albumin: possible involvement in the progression of oxidative stress in hemodialysis patients. Hypertension research: official journal of the Japanese Society of Hypertension 28, 973–980, 10.1291/hypres.28.973 (2005). 16671336

[b12] Rosenkranz A. R. . Novel C5-dependent mechanism of neutrophil stimulation by bioincompatible dialyzer membranes. Journal of the American Society of Nephrology: JASN 10, 128–135 (1999). 9890318 10.1681/ASN.V101128

[b13] Vanholder R., Van Biesen W. & Ringoir S. Contributing factors to the inhibition of phagocytosis in hemodialyzed patients. Kidney international 44, 208–214 (1993). 8355462 10.1038/ki.1993.232

[b14] Leber B., Mayrhauser U., Rybczynski M. & Stadlbauer V. Innate immune dysfunction in acute and chronic liver disease. Wien Klin Wochenschr 121, 732–744, 10.1007/s00508-009-1288-2 (2009). 20047110

[b15] Magnusson M., Magnusson K. E., Sundqvist T. & Denneberg T. Reduced intestinal permeability measured by differently sized polyethylene glycols in acute uremic rats. Nephron 60, 193–198 (1992). 1553004 10.1159/000186738

[b16] Magnusson M., Magnusson K. E., Sundqvist T. & Denneberg T. Impaired intestinal barrier function measured by differently sized polyethylene glycols in patients with chronic renal failure. Gut 32, 754–759 (1991). 1855681 10.1136/gut.32.7.754 PMC1378990

[b17] Magnusson M., Magnusson K. E., Sundqvist T. & Denneberg T. Increased intestinal permeability to differently sized polyethylene glycols in uremic rats: effects of low- and high-protein diets. Nephron 56, 306–311 (1990). 2077413 10.1159/000186158

[b18] Magnusson M., Magnusson K. E., Sundqvist T. & Denneberg T. Urinary excretion of differently sized polyethylene glycols after intravenous administration in uremic and control rats: effects of low- and high-protein diets. Nephron 56, 312–316 (1990). 2077414 10.1159/000186159

[b19] Magnusson M., Magnusson K. E. & Denneberg T. Impaired gut barrier in experimental chronic uremic rats. Mineral and electrolyte metabolism 18, 288–292 (1992). 1465077

[b20] Goncalves S. . Associations between renal function, volume status and endotoxaemia in chronic kidney disease patients. Nephrology, dialysis, transplantation: official publication of the European Dialysis and Transplant Association - European Renal Association 21, 2788–2794, 10.1093/ndt/gfl273 (2006). 16861246

[b21] Khanna A., Rossman J. E., Fung H. L. & Caty M. G. Intestinal and hemodynamic impairment following mesenteric ischemia/reperfusion. J Surg Res 99, 114–119, 10.1006/jsre.2001.6103 (2001). 11421612

[b22] Raj D. S. . Soluble CD14 levels, interleukin 6, and mortality among prevalent hemodialysis patients. American Journal of Kidney Diseases: The Official Journal of the National Kidney Foundation 54, 1072–1080, 10.1053/j.ajkd.2009.06.022 (2009). 19733948 PMC2787958

[b23] Raj D. S., Shah V. O., Rambod M., Kovesdy C. P. & Kalantar-Zadeh K. Association of soluble endotoxin receptor CD14 and mortality among patients undergoing hemodialysis. American Journal of Kidney Diseases: The Official Journal of the National Kidney Foundation 54, 1062–1071, 10.1053/j.ajkd.2009.06.028 (2009). 19699018 PMC3737251

[b24] Wong J., Vilar E. & Farrington K. Endotoxemia in end-stage kidney disease. Semin Dial 28, 59–67, 10.1111/sdi.12280 (2015). 25040340

[b25] Prytz H., Holst-Christensen J., Korner B. & Liehr H. Portal venous and systemic endotoxaemia in patients without liver disease and systemic endotoxaemia in patients with cirrhosis. Scand. J. Gastroenterol. 11, 857–863 (1976). 794999

[b26] Nolan J. P. The role of intestinal endotoxin in liver injury: a long and evolving history. Hepatology 52, 1829–1835, 10.1002/hep.23917 (2010). 20890945

[b27] Hurley J. C. Reappraisal with meta-analysis of bacteremia, endotoxemia, and mortality in gram-negative sepsis. Journal of clinical microbiology 33, 1278–1282 (1995). 7615741 10.1128/jcm.33.5.1278-1282.1995 PMC228145

[b28] Johnson D. W. . Association of dialysis modality and cardiovascular mortality in incident dialysis patients. Clinical journal of the American Society of Nephrology: CJASN 4, 1620–1628, 10.2215/CJN.01750309 (2009). 19729428 PMC2758255

[b29] Aslam N., Bernardini J., Fried L., Burr R. & Piraino B. Comparison of infectious complications between incident hemodialysis and peritoneal dialysis patients. Clinical journal of the American Society of Nephrology: CJASN 1, 1226–1233, 10.2215/CJN.01230406 (2006). 17699352

[b30] Sardenberg C. . Effects of uraemia and dialysis modality on polymorphonuclear cell apoptosis and function. Nephrology, dialysis, transplantation: official publication of the European Dialysis and Transplant Association - European Renal Association 21, 160–165, 10.1093/ndt/gfi095 (2006). 16155068

[b31] Daniels I. . Hydrogen peroxide generation by polymorphonuclear leukocytes exposed to peritoneal dialysis effluent. Clinical and diagnostic laboratory immunology 3, 682–688 (1996). 8914759 10.1128/cdli.3.6.682-688.1996 PMC170431

[b32] Kotanko P., Carter M. & Levin N. W. Intestinal bacterial microflora--a potential source of chronic inflammation in patients with chronic kidney disease. Nephrology, dialysis, transplantation: official publication of the European Dialysis and Transplant Association - European Renal Association 21, 2057–2060, 10.1093/ndt/gfl281 (2006). 16762961

[b33] Simenhoff M. L. . Biomodulation of the toxic and nutritional effects of small bowel bacterial overgrowth in end-stage kidney disease using freeze-dried Lactobacillus acidophilus. Mineral and electrolyte metabolism 22, 92–96 (1996). 8676836

[b34] Goncalves S. . Associations between renal function, volume status and endotoxaemia in chronic kidney disease patients. Nephrol Dial Transplant 21, 2788–2794, 10.1093/ndt/gfl273 (2006). 16861246

[b35] Devolder I., Verleysen A., Vijt D., Vanholder R. & Van Biesen W. Body composition, hydration, and related parameters in hemodialysis versus peritoneal dialysis patients. Perit Dial Int 30, 208–214, 10.3747/pdi.2008.00284 (2010). 20081049

[b36] van Elburg R. M. . Repeatability of the sugar-absorption test, using lactulose and mannitol, for measuring intestinal permeability for sugars. Journal of pediatric gastroenterology and nutrition 20, 184–188 (1995). 7714684 10.1097/00005176-199502000-00008

[b37] Ruan P., Gong Z. J. & Zhang Q. R. Changes of plasma D(-)-lactate, diamine oxidase and endotoxin in patients with liver cirrhosis. Hepatobiliary & pancreatic diseases international: HBPD INT 3, 58–61 (2004). 14969839

[b38] Su Y., Wu S., Fan Y., Jin J. & Zhang Z. The preliminary experimental and clinical study of the relationship between the pigment gallstone and intestinal mucosal barrier. Journal of gastroenterology and hepatology 24, 1451–1456, 10.1111/j.1440-1746.2009.05842.x (2009). 19486450

[b39] Zhang J. . Early gut barrier dysfunction in patients with severe acute pancreatitis: attenuated by continuous blood purification treatment. The International journal of artificial organs 33, 706–715 (2010). 21077043

[b40] Zhang Z. . Intestinal mucosal permeability of children with cefaclor-associated serum sickness-like reactions. European journal of pediatrics 172, 537–543, 10.1007/s00431-012-1926-y (2013). 23296953

[b41] Zhang L., Fan X., Zhong Z., Xu G. & Shen J. Association of plasma diamine oxidase and intestinal fatty acid-binding protein with severity of disease in patient with heat stroke. The American journal of emergency medicine 33, 867–871, 10.1016/j.ajem.2015.01.047 (2015). 25913083

[b42] Kong W. . Biomarkers for assessing mucosal barrier dysfunction induced by chemotherapy: Identifying a rapid and simple biomarker. Clinical laboratory 61, 371–378 (2015). 25975005 10.7754/clin.lab.2014.140712

[b43] Meng Y. . Evaluating Intestinal Permeability by Measuring Plasma Endotoxin and Diamine Oxidase in Children with Acute Lymphoblastic Leukemia Treated with High-dose Methotrexate. Anti-cancer agents in medicinal chemistry 16, 387–392 (2016). 26265099 10.2174/1871520615666150812125955

[b44] Ribitsch W. . Increased Hepato-Splanchnic Vasoconstriction in Diabetics during Regular Hemodialysis. PLoS One 10, e0145411, 10.1371/journal.pone.0145411 (2015). 26713734 PMC4695079

[b45] McIntyre C. W. . Circulating endotoxemia: a novel factor in systemic inflammation and cardiovascular disease in chronic kidney disease. Clinical journal of the American Society of Nephrology: CJASN 6, 133–141, 10.2215/CJN.04610510 (2011). 20876680 PMC3022234

[b46] Vaziri N. D., Zhao Y. Y. & Pahl M. V. Altered intestinal microbial flora and impaired epithelial barrier structure and function in CKD: the nature, mechanisms, consequences and potential treatment. Nephrology, dialysis, transplantation: official publication of the European Dialysis and Transplant Association - European Renal Association, 10.1093/ndt/gfv095 (2015). 25883197

[b47] Stadlbauer V. . Role of Toll-like receptors 2, 4, and 9 in mediating neutrophil dysfunction in alcoholic hepatitis. Am. J. Physiol. Gastrointest. Liver Physiol. 296, G15–G22 (2009). 19033535 10.1152/ajpgi.90512.2008 PMC2636930

[b48] Hernandez M. R. . Biocompatibility of cellulosic and synthetic membranes assessed by leukocyte activation. Am J Nephrol 24, 235–241, 10.1159/000077395 (2004). 15031626

[b49] Ward R. A. & McLeish K. R. Polymorphonuclear leukocyte oxidative burst is enhanced in patients with chronic renal insufficiency. Journal of the American Society of Nephrology: JASN 5, 1697–1702 (1995). 7780059 10.1681/ASN.V591697

[b50] Nakayama M. . Polymorphonuclear leukocyte injury by methylglyoxal and hydrogen peroxide: a possible pathological role for enhanced oxidative stress in chronic kidney disease. Nephrol Dial Transplant 23, 3096–3102, 10.1093/ndt/gfn218 (2008). 18443210

[b51] Kumar S., Bogle R. & Banerjee D. Why do young people with chronic kidney disease die early? World J Nephrol 3, 143–155, 10.5527/wjn.v3.i4.143 (2014). 25374808 PMC4220347

[b52] Carrero J. J. & Stenvinkel P. Inflammation in end-stage renal disease–what have we learned in 10 years? Seminars in dialysis 23, 498–509, 10.1111/j.1525-139X.2010.00784.x (2010). 21039875

[b53] Yoon J. W., Pahl M. V. & Vaziri N. D. Spontaneous leukocyte activation and oxygen-free radical generation in end-stage renal disease. Kidney international 71, 167–172, 10.1038/sj.ki.5002019 (2007). 17136029

[b54] Mera K. . The structure and function of oxidized albumin in hemodialysis patients: Its role in elevated oxidative stress via neutrophil burst. Biochem. Biophys. Res. Commun. 334, 1322–1328 (2005). 16054887 10.1016/j.bbrc.2005.07.035

[b55] Descamps-Latscha B. & Witko-Sarsat V. Importance of oxidatively modified proteins in chronic renal failure. Kidney international. Supplement 78, S108–S113, 10.1046/j.1523-1755.2001.59780108.x (2001). 11168994

[b56] Mafra D. . Role of altered intestinal microbiota in systemic inflammation and cardiovascular disease in chronic kidney disease. Future microbiology 9, 399–410, 10.2217/fmb.13.165 (2014). 24762311

[b57] Mera K. . The structure and function of oxidized albumin in hemodialysis patients: Its role in elevated oxidative stress via neutrophil burst. Biochemical and biophysical research communications 334, 1322–1328, 10.1016/j.bbrc.2005.07.035 (2005). 16054887

[b58] Witko-Sarsat V. . Advanced oxidation protein products as novel mediators of inflammation and monocyte activation in chronic renal failure. Journal of immunology (Baltimore, Md.: 1950) 161, 2524–2532 (1998). 9725252

[b59] Sela S. . Primed peripheral polymorphonuclear leukocyte: a culprit underlying chronic low-grade inflammation and systemic oxidative stress in chronic kidney disease. Journal of the American Society of Nephrology: JASN 16, 2431–2438, 10.1681/asn.2004110929 (2005). 15987755

[b60] Stadlbauer V., Davies N. A., Wright G. & Jalan R. Endotoxin measures in patients’ sample: how valid are the results? Journal of hepatology 47, 726–727; author reply 727-723, 10.1016/j.jhep.2007.08.001 (2007). 17850915

[b61] Bode C., Kugler V. & Bode J. C. Endotoxemia in patients with alcoholic and non-alcoholic cirrhosis and in subjects with no evidence of chronic liver disease following acute alcohol excess. J. Hepatol. 4, 8–14%17 1987/1902/1901%1988 Feb %! Endotoxemia in patients with alcoholic and non-alcoholic cirrhosis and in subjects with no evidence of chronic liver disease following acute alcohol excess %@ 0168-8278 (Print) (1987). 3571935 10.1016/s0168-8278(87)80003-x

[b62] Levin J., Poore T. E., Zauber N. P. & Oser R. S. Detection of endotoxin in the blood of patients with sepsis due to gran-negative bacteria. The New England journal of medicine 283, 1313–1316, 10.1056/NEJM197012102832404 (1970). 5478453

[b63] Novitsky T. J. Limitations of the Limulus amebocyte lysate test in demonstrating circulating lipopolysaccharides. Annals of the New York Academy of Sciences 851, 416–421 (1998). 9668634 10.1111/j.1749-6632.1998.tb09018.x

[b64] Horvath A. . Randomised clinical trial: the effects of a multispecies probiotic vs. placebo on innate immune function, bacterial translocation and gut permeability in patients with cirrhosis. Aliment. Pharmacol. Ther., n/a-n/a, 10.1111/apt.13788 (2016). PMC5053220 27593544

[b65] Furst W. & Hallstrom S. Simultaneous determination of myocardial nucleotides, nucleosides, purine bases and creatine phosphate by ion-pair high-performance liquid chromatography. J. Chromatogr. 578, 39–44 (1992). 1400784 10.1016/0378-4347(92)80222-c

[b66] Hallstrom S. . S-nitroso human serum albumin treatment reduces ischemia/reperfusion injury in skeletal muscle via nitric oxide release. Circulation 105, 3032–3038 (2002). 12081999 10.1161/01.cir.0000018745.11739.9b

[b67] Pelzmann B. . NADH supplementation decreases pinacidil-primed I K ATP in ventricular cardiomyocytes by increasing intracellular ATP. Br. J. Pharmacol. 139, 749–754 (2003). 12812998 10.1038/sj.bjp.0705300 PMC1573896

[b68] Marsche G. . Plasma-advanced oxidation protein products are potent high-density lipoprotein receptor antagonists *in vivo*. Circulation research 104, 750–757, 10.1161/CIRCRESAHA.108.193169 (2009). 19179658 PMC3375477

[b69] Holzer M. . Aging affects high-density lipoprotein composition and function. Biochimica et biophysica acta 1831, 1442–1448, 10.1016/j.bbalip.2013.06.004 (2013). 23792422 PMC3787738

[b70] Kawai K. . Effect of diabetic retinopathy on redox state of aqueous humor and serum albumin in patients with senile cataract. Tokai J. Exp. Clin. Med. 26, 93–99 (2001). 11885755

